# Phase II study of apatinib in combination with oral vinorelbine in heavily pretreated HER2-negative metastatic breast cancer and clinical implications of monitoring ctDNA

**DOI:** 10.20892/j.issn.2095-3941.2020.0418

**Published:** 2021-08-15

**Authors:** Anjie Zhu, Peng Yuan, Nanlin Hu, Mingzhou Li, Wenmiao Wang, Xue Wang, Jian Yue, Jiayu Wang, Yang Luo, Fei Ma, Pin Zhang, Qing Li, Binghe Xu, Shanbo Cao, Giuseppe Lippi, Yoichi Naito, Mohammed A. Osman, Gustavo N. Marta, Gianluca Franceschini, Armando Orlandi

**Affiliations:** 1 Department of Medical Oncology, National Cancer Center/National Clinical Research Center for Cancer/Cancer Hospital, Chinese Academy of Medical Sciences and Peking Union Medical College, Beijing 100021, China; 2 Key Laboratory of Carcinogenesis and Translational Research (Ministry of Education/Beijing), Department of Breast Oncology, Peking University Cancer Hospital & Institute, Beijing 100142, China; 3 Department of VIP Medical Services, National Cancer Center/National Clinical Research Center for Cancer/Cancer Hospital, Chinese Academy of Medical Sciences and Peking Union Medical College, Beijing 100021, China; 4 Department of Pathology, National Cancer Center/National Clinical Research Center for Cancer/Cancer Hospital, Chinese Academy of Medical Sciences and Peking Union Medical College, Beijing 100021, China; 5 AcornMed Biotechnology Co., Ltd., Beijing 101102, China; 6 Section of Clinical Biochemistry, University Hospital of Verona, Verona 37100, Italy; 7 Department of Breast and Medical Oncology, National Cancer Center Hospital East, Kashiwa 277-8577, Japan; 8 Clinical Oncology, General Organization for Teaching Hospitals, Cairo 11435, Egypt; 9 Department of Radiation Oncology, Hospital Sírio-Libanês, Sao Paulo 01308-050, Brazil; 10 Multidisciplinary Breast Center, Fondazione Policlinico Universitario A. Gemelli IRCCS, Università Cattolica del Sacro Cuore, Rome 00176, Italy; 11 Unit of Medical Oncology, Fondazione Policlinico Universitario A. Gemelli IRCCS, Roma 00176, Italy

**Keywords:** Metastatic breast cancer, apatinib, oral vinorelbine, ctDNA

## Abstract

**Objective:**

Apatinib is an oral TKI targeting VEGFR-2. Single-agent apatinib treatment has been shown to produce an objective response in patients with pretreated mBC. Oral vinorelbine also holds promise as a treatment of choice in patients with mBC. This study aimed to investigate the efficacy and safety of the oral vinorelbine-apatinib combination in patients with pretreated mBC. In addition, we detected gene variants in ctDNA to explore the therapeutic implications.

**Methods:**

This study enrolled patients with HER2-negative mBC who were pretreated with anthracycline/taxanes. Patients were treated with apatinib at 500 mg/425 mg daily plus oral vinorelbine 60 mg/m^2^ on days 1, 8, and 15 of every cycle (3 weeks). The primary endpoint was PFS. The secondary endpoints were ORR, CBR, OS, and safety. Patients eligible for ctDNA detection were evaluated before and during treatment.

**Results:**

Forty patients were enrolled. The median PFS was 5.2 months (95% CI, 3.4–7.0 months), and the median OS was 17.4 months (95% CI, 8.0–27.0 months). The ORR was 17.1% (6/35), and the CBR was 45.7% (16/35). The most common AEs included gastrointestinal reaction, myelosuppression, and hypertension. In 20 patients, ctDNA was detected at baseline and during treatment. A significant difference was found in PFS for undetected *vs.* detected baseline ctDNA (13.9 months *vs.* 3.6 months, *P* = 0.018).

**Conclusions:**

All-oral therapy with apatinib plus vinorelbine displayed objective efficacy in patients with heavily pretreated HER2-negative mBC, with acceptable and manageable toxicity profiles. Patients with no gene variant detected and lower variant allele frequencies in ctDNA at baseline showed longer PFS.

## Introduction

Despite a recent decline in breast cancer (BC) mortality, metastatic BC (mBC) remains an incurable disease^1^. Angiogenesis is an important factor in tumor growth, invasion, and metastasis^2^. Vascular endothelial growth factor (VEGF) and its receptor, vascular endothelial growth factor receptor (VEGFR), are key factors regulating neovascularization^3^. Studies on antiangiogenic agents have continued to develop applications in the treatment of mBC. Although prolonged progression-free survival (PFS) has been observed with chemotherapy, the lack of improvement in overall survival (OS) along with the presence of severe adverse events (AEs) has limited the application of antiangiogenic agents^4–11^.

Apatinib is a novel small-molecule oral tyrosine kinase inhibitor that selectively binds VEGFR-2, thus decreasing VEGF-mediated endothelial cell migration, proliferation, and tumor microvessel density^12^. Apatinib, an inhibitor of VEGFR-2 through selective competition for ATP binding sites, is more specific to VEGFR-2^13^. Preclinical studies suggest that apatinib can reverse P-glycoprotein (P-gp/ABCB1)- and BC resistance protein (BCRP/ABCG2)-mediated multidrug resistance, thus amplifying the cytotoxicity of chemotherapeutic drugs such as anthracyclines, taxanes, and vinca alkaloids^14,15^. Two multicenter phase II studies have reported an objective response rate (ORR) of apatinib in mBC treatment of 0.7%–16.7%, a median PFS of 3.3–4.0 months, and a median OS of 10.3–10.6 months^16,17^. On the basis of preclinical and clinical data, researchers have become increasingly interested in evaluating the efficacy of apatinib combined with chemotherapy in patients with mBC^18–20^.

Vinorelbine is a semisynthetic vinca alkaloid antitumor agent. Single-agent or combination chemotherapy with vinorelbine has shown efficacy in patients with mBC. The ORR of oral single-agent regimens in the first-line treatment of metastatic HER2-negative BC has been reported to be 11%–31%^21–24^ and to reach 50%–60% when these regimens are combined with agents such as capecitabine^25–29^. In addition, vinorelbine exhibits anti-angiogenic properties^30^. For low-dose chemotherapy combined with anti-angiogenesis, Klement et al.^31^ have validated the rationale in which any anti-vascular effects of the low-dose chemotherapy would be selectively enhanced when survival signals mediated by VEGF were inhibited. Preclinical and clinical studies have revealed that anti-angiogenetic agents and vinorelbine have synergistic anti-tumor functions in NSCLC and BC^32–35^. On the basis of this evidence, and given the relatively benign AE profile and convenience of administration, we selected oral vinorelbine as a combination agent to be used with the antiangiogenic tyrosine kinase inhibitor (TKI) apatinib.

Analysis of circulating tumor DNA (ctDNA) is a widespread method of liquid biopsy, which enables disease diagnosis^36,37^, prognostication^38,39^, detection of recurrence^40,41^, monitoring of tumor burden, and identification of therapeutic responses^42^ and resistance^43,44^ in patients with BC.

This phase II study aimed to prospectively explore the efficacy and safety of apatinib combined with oral vinorelbine in patients with metastatic HER2-negative BC. This study further detected somatic mutations in plasma ctDNA at baseline and during treatment in patients treated with apatinib plus oral vinorelbine, to explore the potential association between ctDNA and clinical outcomes.

## Materials and methods

### Patients and methods

#### Ethical approval

The present study was approved by the National Cancer Center/National Clinical Research Center for Cancer/Cancer Hospital, Chinese Academy of Medical Sciences and Peking Union Medical College (Approval No. CH-BC-046) and registered at clinicaltrials.gov (No. NCT02768415). Written informed consent to participate in the study was obtained from all patients or their legal guardians. The study was performed in accordance with the relevant guidelines and regulations.

#### Included patients

The study population included patients with HER2-negative mBC for whom previous treatment failed. The specific inclusion criteria and exclusion criteria are described in **Supplementary material 1**.

#### Therapeutic protocol

The initial oral dose of apatinib was 500 mg/day with 21 days/cycle. Investigators could adjust the initial dose down to 425 mg/day according to age, ECOG score, and body surface area. If patients had grade 3/4 hematologic AEs, hypertension, proteinuria, hand-foot skin reaction, mucositis, or grade 3/4 nonhematologic AEs requiring intervention, delayed administration and dose adjustment were considered: if the initial dose was 500 mg/day, then it was reduced to 425 mg/day for the first dose and 250 mg/day for the second dose; if the initial dose was 425 mg/day, it was reduced to 250 mg/day. Apatinib was taken continuously until disease progression, intolerance of AEs after dose modification, withdrawal of informed consent, or administration delays of > 14 days due to toxicity.

Oral vinorelbine 60 mg/m^2^ (body surface area) was administered on days 1, 8, and 15 of the 21-day cycle. If patients had grade 3/4 hematologic AEs or grade 3/4 nonhematologic AEs that required intervention, delayed administration and dose adjustment were considered. Vinorelbine was taken continuously until disease progression, intolerance to AEs after dose adjustment, withdrawal of informed consent, or administration delays of > 21 days due to toxicity.

#### Study design

This was a single-arm, open-label phase II study. The primary endpoint was PFS. The secondary endpoints were ORR, clinical benefit rate (CBR), OS, and safety. PFS was defined as the time from registration to the date of disease progression or death from any cause. OS was defined as the time from registration to the date of death from any cause or the last follow-up visit. Efficacy was evaluated every 2 cycles until disease progression, intolerable AEs, withdrawal of informed consent, or delayed dosing beyond the prescribed period. Delayed administration was defined as failure to take the drug on time for ≥ 3 days during treatment.

According to the response evaluation criteria in solid tumors (RECIST) 1.1, efficacy was categorized as complete response (CR), partial response (PR), stable disease (SD), or progressive disease (PD). ORR was defined as the proportion of eligible patients who achieved confirmed CR or PR. CBR was defined as the proportion of patients who achieved CR, PR, or SD for at least 24 weeks.

AEs were assessed and graded in accordance with the common terminology criteria for adverse events (CTCAE) 4.0.

### ctDNA detection

Patients who consented to at least one blood draw were eligible for further ctDNA analysis and constituted the study population. Library preparation, NGS sequencing, and bioinformatics analysis were performed as described in **Supplementary material 2**.

### Statistical analysis

Previously reported data have indicated that the median PFS with oral vinorelbine combination with capecitabine as a second-line treatment for metastatic HER2-negative BC is 3.8 months^45^. The study was designed to be two-sided, with an α-error of 5% and a power of 80%. We expected that the median PFS for patients receiving apatinib combined with oral vinorelbine would be 6 months. Assuming a 15% dropout rate and 6-month follow-up period, the final accrual number was 40.

Data were summarized as frequency and percentage for qualitative variables, and as medians and ranges for quantitative variables. The PFS and OS were calculated from the date of registration to the first documented date of disease progression and date of death, respectively, with the Kaplan-Meier method. Associated 95% CIs were calculated with the Brookmeyer-Crowley method. The log-rank test was used for comparison of PFS and OS between groups. The multiple Cox model was used to evaluate significant differences in PFS and OS between groups. Statistical analyses were conducted with SPSS 21.0 statistical software (SPSS, Chicago, IL, USA).

## Results

### Patient information

The baseline patient characteristics are shown in **Table 1**. Forty patients with HER2-negative mBC were enrolled at our institution between May 2016 and January 2018 (median age, 55 years; range 30–70 years). Twenty-one patients (52.5%) received apatinib combined with oral vinorelbine as a second-line treatment, and 19 patients (47.5%) received it as a third-line treatment or beyond. All 40 patients were included in the survival and safety analyses. Five patients were discharged before the first efficacy evaluation. A total of 35 patients were included in the efficacy evaluation analysis. At the time of the final follow-up (November 30, 2019), 33 (82.5%) patients had disease progression, and 28 (70.0%) patients had died. Twenty-seven (67.5%) patients had delayed administration of apatinib or oral vinorelbine during treatment. Twenty-one (52.5%) patients experienced apatinib or oral vinorelbine dose modification.

**Table 1:** Patient characteristics at baseline

Characteristics	*n* (%)
Age (years)	
< 55	20 (50.0)
≥ 55	20 (50.0)
ECOG performance status	
0	27 (67.5)
1	13 (32.5)
Hormone receptor	
Negative	20 (50.0)
Positive	20 (50.0)
Histopathologic grade	
I–II	18 (45.0)
III	16 (40.0)
Unknown	6 (15.0)
Tumor size (cm)	
≤ 2.0	13 (32.5)
> 2.0	21 (52.5)
Unknown	6 (15.0)
Axillary lymph node metastasis	
Positive	29 (72.5)
Negative	7 (17.5)
Unknown	4 (10.0)
TNM stage at diagnosis	
I–II	15 (37.5)
III	18 (45.0)
Unknown	7 (17.5)
Local recurrence	
Regional lymph node	20 (50.0)
Chest wall	17 (42.5)
Distant metastasis	
Distant lymph node	20 (50.0)
Bone	19 (47.5)
Lung	13 (32.5)
Liver	10 (25.0)
Pleura	4 (10.0)
Skin	3 (7.5)
Brain	2 (5.0)
Metastasis ≥ 3 sites	21 (52.5)
Starting dose of apatinib	
425 mg	23 (57.5)
500 mg	17 (42.5)
Lines of apatinib plus vinorelbine treatment	
< 3 line	21 (52.5)
≥ 3 line	19 (47.5)

### Safety

No treatment-related deaths occurred, but 28 patients died because of disease progression.

The initial dose of apatinib was 500 mg/day for the first 17 patients. During the first cycle of treatment, 6 patients (35.3%) developed grade 3 hypertension with poor control from the combined antihypertensive therapy, 1 patient (5.9%) had grade 3 proteinuria, 1 patient (5.9%) developed a grade 3 hand-foot skin reaction, and 1 patient (5.9%) was hospitalized for grade 3 symptomatic thrombocytopenia. Because of tolerance concerns, all patients enrolled after the 17th patient were given a lower initial dose of 425 mg/day. Among patients who underwent dose modification with apatinib (*n* = 8), 5 had a final dose of 425 mg/day, and 3 had a final dose of 250 mg/day. Twenty-three patients had an initial apatinib dose of 425 mg/day. No significant correlation was found between the initial apatinib dose and the onset of delayed administration or grade 3/4 AEs. The specifics of the initial dose, delayed administration, and dose modification of apatinib are shown in **Supplementary Table S2**.

Most AEs were grade 1–2 and were well tolerated (**Table 2**). The most common AEs included gastrointestinal reaction, myelosuppression, and hypertension. One patient was hospitalized for grade 3 symptomatic thrombocytopenia. Among the patients with an initial apatinib dose of 500 mg/day, the incidence of grade 3/4 AEs was 58.8%, which was higher than that in the patients with an initial dose of 425 mg/day (43.5%) (**Supplementary Table S3**).

**Table 2:** Summary of adverse events

Adverse events, *n* = 40	All grades, *n* (%)	Grade 1/2, *n* (%)	Grade 3/4, *n* (%)
Myelosuppression (hematology)	27 (67.5)	21 (52.5)	6 (15.0)
Leukopenia	22 (55.0)	20 (50.0)	2 (5.0)
Neutropenia	22 (55.0)	17 (42.5)	5 (12.5)
Thrombocytopenia	10 (25.0)	9 (22.5)	1 (2.5)
Decreased hemoglobin	9 (22.5)	9 (22.5)	0 (0)
Gastrointestinal reaction	28 (70.0)	19 (47.5)	9 (22.5)
Nausea	23 (57.5)	22 (55.0)	1 (2.5)
Diarrhea	19 (47.5)	13 (32.5)	6 (15.0)
Vomiting	12 (30.0)	10 (25.0)	2 (5.0)
Hypertension	25 (62.5)	15 (37.5)	10 (25.0)
Pain	24 (60.0)	19 (47.5)	5 (12.5)
Malaise	21 (52.5)	19 (47.5)	2 (5.0)
Anorexia	20 (50.0)	19 (47.5)	1 (2.5)
Elevated transaminase	19 (47.5)	19 (47.5)	0 (0)
Hand-foot skin reaction	19 (47.5)	16 (40.0)	3 (7.5)
Proteinuria	15 (37.5)	14 (35.0)	1 (2.5)
Elevated bilirubin	13 (32.5)	12 (30.0)	1 (2.5)
Mucositis	11 (27.5)	8 (20.0)	3 (7.5)
Hemorrhage	8 (20.0)	7 (17.5)	1 (2.5)
Sinus tachycardia	6 (15.0)	6 (15.0)	0 (0)
Elevated creatinine	3 (7.5)	3 (7.5)	0 (0)

The main drug-related specific AEs, the median time of first delayed administration, and the median time of first dose modification were analyzed from the initiation of combined treatment (**Supplementary Table S4**).

### Efficacy

**Figure 1:**
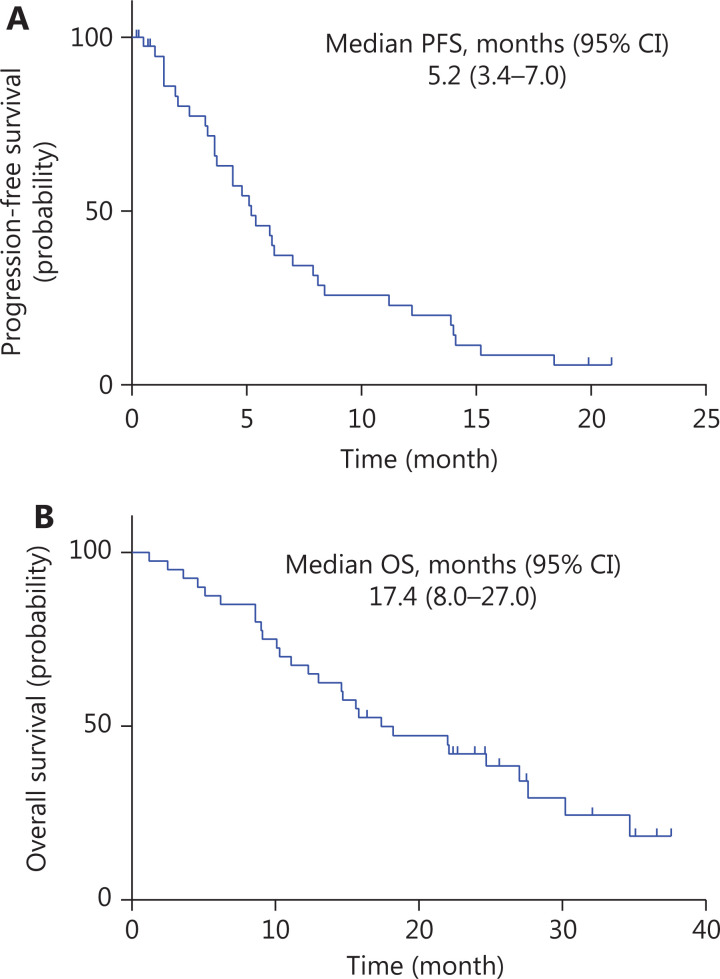
Kaplan-Meier curve of progression-free survival (PFS) and overall survival (OS) in patients with pretreated advanced breast cancer who received apatinib combined therapy. (A) Kaplan-Meier curve of PFS, indicating a median PFS of 5.2 months (95% CI: 3.4–7.0); (B) Kaplan-Meier curve of OS, indicating a median OS of 17.4 months (95% CI: 8.0–27.0).

Thirty-three patients had disease progression, and 28 patients died. The median PFS was 5.2 months (95% CI, 3.4–7.0 months, **Figure 1A**), and the median OS was 17.4 months (95% CI, 8.0–27.0 months, **Figure 1B**). Among the 35 patients with evaluable efficacy, 6 (17.1%) achieved a better response to PR, 23 (65.7%) achieved SD, and the ORR was 17.1% (6/35). Sixteen patients achieved PR or remained in SD for > 24 weeks, and the CBR was 45.7% (16/35). The efficacy by number of treatment lines and HR expression is shown in **Supplementary Table S5**.

**Figure 2:**
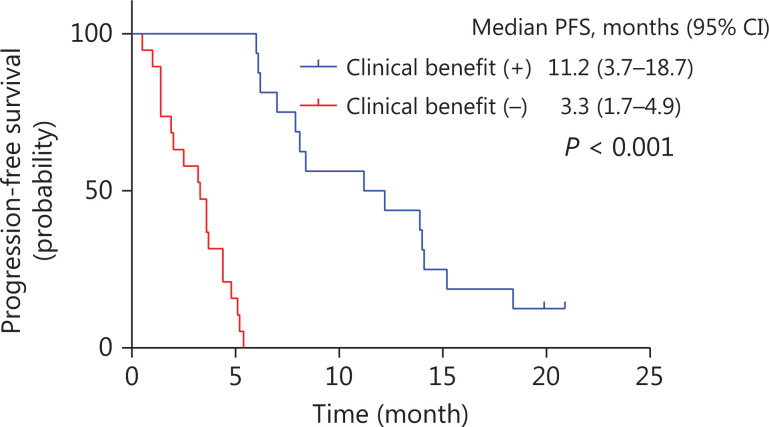
Kaplan-Meier curve of PFS comparing patients who achieved a clinical benefit after apatinib combined therapy, with a median PFS of 11.2 months, and those who did not, with a median PFS of 3.3 months. A statistically significant difference was found between these patient groups (*P* < 0.001).

The median PFS was significantly longer for patients who showed clinical benefit (i.e., CR, PR, or SD for at least 24 weeks) (*n* = 16) than for those who did not (*n* = 19) [11.2 months (95% CI, 3.7–18.7 months) *vs.* 3.3 months (95% CI, 1.7–4.9 months), respectively, *P* < 0.001, **Figure 2**]. Nevertheless, no significant OS difference was found between these patient groups (*P* = 0.271).

**Figure 3:**
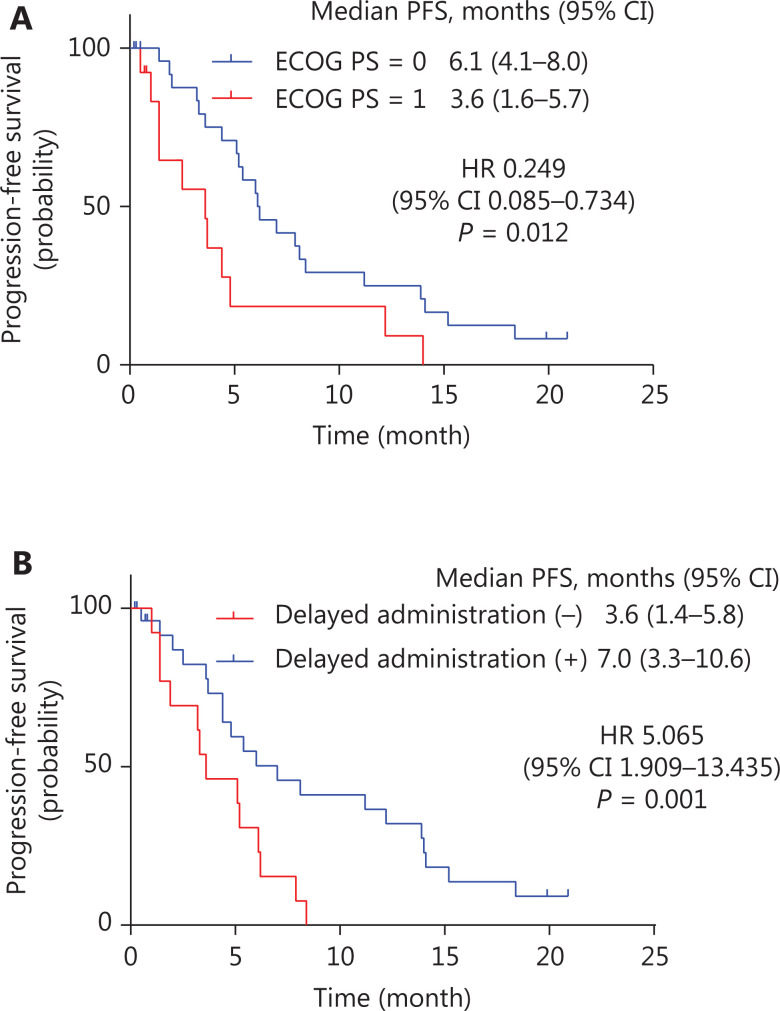
Kaplan-Meier curve of progression-free survival (PFS) with statistical significance in subgroups. (A) Kaplan-Meier curve of PFS comparing patients whose ECOG PS score was 0, with a median PFS of 6.1 months (95% CI: 4.1–8.0), and those whose ECOG PS score was 1, with a median PFS of 3.6 months (95% CI: 1.6–5.7). A statistically significant difference was found between these patient groups (*P* = 0.022); (B) Kaplan-Meier curve of PFS comparing patients who experienced administration delay, with a median PFS of 7.0 months (95% CI: 3.3–10.6), and those who did not, with a median PFS of 3.6 months (95% CI: 1.4–5.8). A statistically significant difference was found between these patient groups (*P* = 0.008).

In the survival analysis, univariate analysis of the correlation between the characteristics and treatment-related conditions and PFS/OS was performed (**Table 3**). The results showed significant prolongation of median PFS in patients with an ECOG PS score of 0 (*P* = 0.022) and delayed administration during treatment (*P* = 0.008) (**Figure 3A and 3B**).

**Table 3:** Subgroup analysis comparing median PFS and OS among patients with different characteristics

Variables	PFS		OS	
	Median PFS (months, 95% CI)	*P*	Median OS (months, 95% CI)	*P*
Age, years				
< 55	4.4 (3.0–5.8)	0.159	24.7 (10.3–39.0)	0.408
≥ 55	6.2 (2.5–9.9)		15.6 (10.0–21.3)	
ECOG performance status				
0	6.1 (4.1–8.0)	0.022	22.0 (14.0–30.0)	0.242
1	3.6 (1.6–5.7)		9.1 (1.9–16.2)	
Hormone receptor status				
Positive	4.4 (2.0–6.8)	0.194	18.2 (0.0–37.9)	0.744
Negative	6.0 (4.1–7.8)		15.8 (11.9–19.7)	
Visceral metastasis				
No	4.8 (2.7–7.0)	0.579	22.0 (9.6–34.3)	0.884
Yes	5.2 (3.2–7.2)		17.4 (10.0–24.8)	
Chest wall recurrence				
No	5.2 (3.2–7.2)	0.98	30.2 (16.7–43.7)	0.036
Yes	4.8 (2.7–7.0)		14.7 (7.6–21.8)	
Number of metastatic sites				
< 3	5.2 (4.6–5.8)	0.684	22.1 (9.2–35.1)	0.816
≥ 3	4.4 (1.1–7.7)		17.4 (10.0–24.8)	
Lines of treatment				
< 3	6.1 (3.4–8.7)	0.875	17.4 (12.3–22.5)	0.734
≥ 3	5.2 (4.0–6.4)		24.7 (11.2–38.1)	
Initial dose of apatinib				
425 mg/day	3.7 (2.6–4.9)	0.100	14.7 (5.2–24.2)	0.542
500 mg/day	7.0 (3.5–10.5)		22.0 (13.5–30.5)	
Hypertension				
No	4.4 (2.5–6.3)	0.587	15.6 (1.8–29.5)	0.563
Yes	5.4 (4.0–6.8)		18.2 (8.2–28.3)	
Hand-foot skin reaction				
No	5.1 (3.7–6.4)	0.951	14.7 (7.7–21.7)	0.516
Yes	5.4 (3.2–7.5)		22.0 (15.3–28.7)	
Proteinuria				
No	4.4 (2.6–6.1)	0.271	14.7 (10.4–18.9)	0.555
Yes	8.1 (5.2–11.0)		22.1 (13.9–30.4)	
Grade 3/4 adverse event				
No	5.1 (1.7–8.4)	0.361	17.4 (4.7–30.2)	0.414
Yes	5.4 (3.3–7.5)		14.7 (6.8–22.6)	
Delayed administration				
No	3.6 (1.4–5.8)	0.008	15.8 (2.4–29.2)	0.728
Yes	7.0 (3.3–10.6)		18.2 (8.1–28.4)	

PFS, progression-free survival; OS, overall survival.

Variables including age, hormone receptor, ECOG PS, apatinib initial dose, proteinuria, and delayed administration were included in multivariate Cox proportional hazard models predicting PFS. An ECOG PS score of 0 and delayed administration remained independent predictive factors of PFS (**Figure 3A, 3B, Table 4**). In addition, chest wall recurrence was an independent predictor of OS (*P* = 0.025) (**Supplementary Table S6**).

**Table 4:** Multivariate Cox proportional hazard models predicting PFS for patients receiving combined therapy

Variables	HR (95% CI)	*P* value
Age (< 55/≥ 55)	1.106 (0.513–2.383)	0.798
Hormone receptor (neg/pos)	0.716 (0.320–1.604)	0.417
ECOG PS (0/1)	0.249 (0.085–0.734)	0.012
Apatinib initial dose (425 mg/500 mg)	0.838 (0.306–2.297)	0.732
Proteinuria (no/yes)	1.349 (0.596–3.053)	0.472
Delayed administration (no/yes)	5.065 (1.909–13.435)	0.001

PFS, progression free survival; ECOG PS, Eastern Cooperative Oncology Group Performance Statues.

### Patient characteristics for ctDNA analysis and detection of mutation profiling in ctDNA

**Figure 4:**
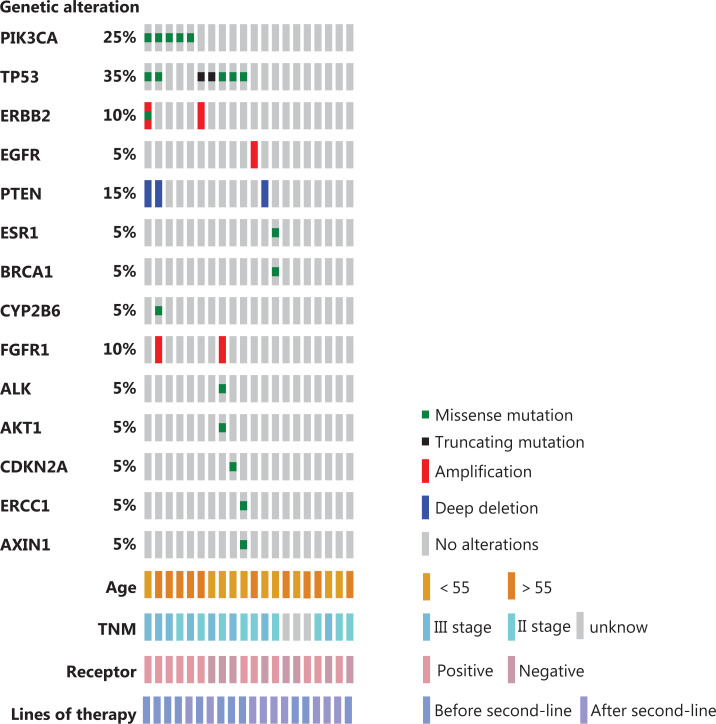
Distribution of the main genomic alterations in the entire population at baseline.

Twenty patients had their first blood drawn at baseline (**Supplementary Table S7**). A total of 52 blood samples were collected, and 57 variant alterations were detected. The most frequently altered genes at baseline were *TP53* (35%), *PIK3CA* (25%), *PTEN* (15%), *ERBB2* (10%), and *FGFR1* (10%). The distribution of the main genomic alterations is shown in **Figure 4**. Specific genomic alterations at baseline and serial monitoring of alterations in ctDNA are shown in **Supplementary Table S8**.

### The associations between baseline gene variants and PFS/OS

**Figure 5:**
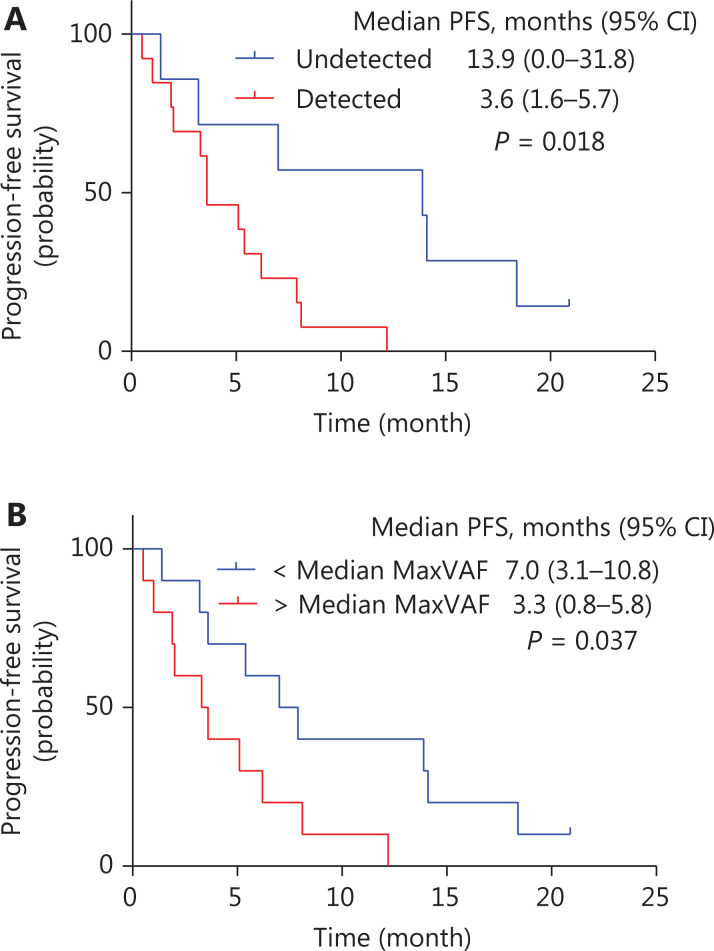
Kaplan-Meier curve of progression-free survival (PFS) in subgroups with ctDNA at baseline. (A) Kaplan-Meier curve of PFS comparing patients with detected gene variants and those without gene variants in ctDNA at baseline. The median PFS was significantly longer for patients who had no gene variants detected in ctDNA compared with patients who had detectable gene variants [13.9 months (95% CI, 0.0–31.8 months) *vs.* 3.6 months (95% CI, 1.6–5.7 months), *P* = 0.018]. (B) Kaplan-Meier curve of PFS comparing patients with different values of maxVAF in ctDNA. Patients whose maxVAF was less than the median value (0.985%) achieved longer PFS than those with higher maxVAF [7.0 months (95% CI, 3.1–10.8 months) *vs.* 3.3 months (95% CI, 0.8–5.8 months), *P* = 0.037].

In analysis of genomic alterations in ctDNA at baseline, the median PFS was significantly longer for patients without detectable gene variants in ctDNA [13.9 months (95% CI, 0.0–31.8 months) *vs.* 3.6 months (95% CI, 1.6–5.7 months), *P* = 0.018, **Figure 5A**]. The median value of the maximum variant allele frequency (maxVAF) was used as the cutoff value (0.985%). Patients whose maxVAF was less than the median value achieved longer PFS than those with higher maxVAF [7.0 months (95% CI, 3.1–10.8 months) *vs.* 3.3 months (95% CI, 0.8–5.8 months), *P* = 0.037, **Figure 5B**]. No statistically significant difference was found in the analysis of OS.

### Dynamic changes in ctDNA gene alterations during follow-up

A total of 12 patients had blood drawn at baseline, and at least one blood sample was collected at the subsequent time points. Four patients were free from genomic alterations at baseline and during treatment, whereas 8 patients displayed genomic alterations in ctDNA. The entire follow-up of these patients and the patterns of changes in ctDNA are shown in **Supplementary Figure S1**.

## Discussion

This is the first study exploring the efficacy and safety of apatinib combined with oral vinorelbine for the treatment of mBC. In this study, nearly half the patients (19/40, 47.5%) had already received at least 2-line metastatic treatment.

The patients enrolled in our study were all HER2-negative. Notwithstanding the limits imposed by comparing different studies, our subgroup analysis of patients with TNBC showed better efficacy than that of the single-agent treatment in Hu’s study^17^. The median PFS and OS of HR-positive patients were also longer in our study than in a non-TNBC apatinib single-agent study^16^, although a discrepancy in the molecular subtypes of these 2 studies makes the studies poorly comparable. Most previous studies of vinorelbine combined with chemotherapy (mainly capecitabine) have focused on first-line therapy for advanced BC. A recent pooled analysis for stage II–III clinical trials of oral or intravenous vinorelbine plus capecitabine^45^ has suggested that combined therapy for second-line treatment has an ORR of 41.0%, a median PFS of 3.8 months, and a median OS of 11.3 months. All our patients received apatinib plus oral vinorelbine as a second-line treatment or beyond, and our data showed that the median PFS and OS for second-line treatment were 4.8 months and 14.7 months, respectively, with an ORR of 20.0%. Compared with the efficacy of previous vinorelbine combined chemotherapy, the PFS and OS for our combination therapy as a second-line treatment were both longer, although the ORR did not improve. A recent Chinese randomized clinical trial comparing vinorelbine and eribulin mesylate has reported a median PFS and median OS for vinorelbine of 2.8 months and 12.5 months in patients with locally recurrent BC or mBC who had at least 2 prior regimens^46^, whereas our results in patients who had received at least 2 regimens showed longer PFS and OS (5.2 months and 27.0 months, respectively). Because PFS and OS are the main objectives in clinical practice, apatinib plus oral vinorelbine as a second-line treatment for mBC may offer better disease control. Simultaneously, our combined therapy may be considered in patients with at least 2 prior regimens.

Beyond its anticancer activity against angiogenesis, apatinib was found to reverse multidrug resistance by decreasing expression of P-glycoprotein (ABCB1) and BC resistance protein (ABCG2) *in vitro*. It also effectively enhanced the drug susceptibility of drug-resistant cell lines. Further studies have demonstrated that a certain concentration of apatinib significantly increases the toxicity of chemotherapy agents such as taxane and vinca alkaloids^14,15^. On the basis of the knowledge regarding the antitumor mechanism and the potential efficacy of apatinib combined with chemotherapy gained from this study, we conclude that synergistic or additive effects may exist between apatinib and chemotherapy. The combination of apatinib and oral vinorelbine may be a promising treatment after failure of other therapies for advanced BC. Therefore, the development of therapies in subsequent research is highly important.

The major AEs in our study were gastrointestinal reaction, myelosuppression, hypertension, pain, malaise, anorexia, elevated transaminase, and hand-foot skin reaction (incidence > 40%). The more frequently observed severe AEs (hypertension, diarrhea, and neutropenia) were reversed after symptomatic treatment, delayed administration, or dose modification, thus suggesting that collecting patient information before treatment may be advisable. The incidence of grade 3–4 AEs was decreased by downregulation of the initial dosage of apatinib in late-enrolled patients, but no significant difference in efficacy between these dose groups was noted. Therefore, we recommend an initial dose of apatinib of 425 mg/day in combination therapy.

Our study also showed that patients who gained clinical benefit after combination therapy achieved longer PFS, thus implying that an ideal short-term outcome predicts good disease control. Huang et al.^47^ have found that the response to apatinib is significantly associated with clinical outcomes in advanced gastric cancer, a finding consistent with our data.

Our study found that patients with an ECOG PS score of 0 had longer PFS, thereby suggesting that patients in good condition are more likely to benefit. Previous studies on the treatment of solid tumors have shown that patients with ECOG PS scores of ≥ 2 often have poorer outcomes^48,49^. Therefore, our results confirm that patients with better health assessment scores would benefit more, thus indicating that patient health condition is essential in evaluating treatment choices^50^.

Our multivariate analysis additionally showed that the treatment schedule may affect PFS. For example, patients with at least one delayed administration had significantly longer PFS than those who strictly followed the schedule. The leading cause of delayed administration was the onset of AEs, and hence the duration of treatment was longer for these patients. The efficacy and AEs may simultaneously increase in patients with higher blood concentrations, whereas drug susceptibility differs because of different targets among individuals. Given the potential therapeutic value of apatinib combined with oral vinorelbine, the optimal administration pattern for improving compliance and efficacy should be further investigated. Although our study did not find a significant difference in PFS or OS between HR-positive and HR-negative patients, the efficacy results suggested a significantly improved ORR in patients with TNBC compared with HR-positive individuals. Microvessel density is an important biomarker of tumor angiogenesis^51,52^, and high expression of VEGF/VEGFR is associated with greater vascular density^53^. Previous studies have found increased tumor VEGF levels in TNBC^54^. However, VEGFR, particularly VEGFR2, is simultaneously expressed with VEGF on the tumor endothelium^53^. Therefore, tumor microvessel density might increase in TNBC with high expression of VEGF/VEGFR, thus leading to a greater benefit from antivascular therapy. However, larger samples of clinical studies are needed to validate this analysis.

In the current study, we also explored the roles of ctDNA variants during antiangiogenic combination therapy. PFS was relatively shorter in patients with more mutations/variants or with higher frequencies of ctDNA alterations. Recently, Rossi et al.^55^ have longitudinally detected ctDNA in patients with mBC, in whom the number of mutations in ctDNA along with the maximum mutant allele fraction at baseline were both predictive of progression and death. Dawson et al.^42^ have confirmed that increasing levels of ctDNA are significantly associated with poorer outcomes. Regarding the factors predicting the efficacy of antiangiogenic-based therapy, no efficient biomarkers have been identified to date. Most studies have focused on the plasma or tissue levels of specific protein expression and single nucleotide polymorphisms in the VEGF signaling pathway. Nevertheless, all these biomarkers lack validation in further clinical studies. For the exploration of gene mutations, a single-arm, phase II study of apatinib in refractory metastatic colorectal cancer has analyzed a panel of 1,021 cancer-related genes by ctDNA, but has not found any positive results associated with PFS or OS^56^. One possible explanation is that by blocking VEGF signal transduction, antiangiogenic therapies act not only on tumor cells but also on the microenvironment^57,58^, thus making identification of a biomarker in ctDNA difficult. Our investigation revealed an association between gene alterations in ctDNA at baseline and outcomes during antiangiogenic-based therapy, thus demonstrating that clinical outcomes depend on somatic variants, in terms of both mutation burden and frequency. However, the association between tumor mutation burden and therapeutic effects is more frequently discussed in immune therapies. Our exploration of ctDNA was based on a single arm study with a small sample size. The nature of the study (e.g., the absence of a control arm) did not enable clarification of the predictive or prognostic role of ctDNA. Larger randomized controlled double-blind clinical trials should be conducted to identify possible biomarkers for effective prediction.

Despite the limited data for the patients with ctDNA examined and the impossibility of distinguishing the predictive or prognostic role, some results of the exploratory analysis deserve further discussion. One patient originally treated with letrozole had an *ESR1* mutation at baseline before combined treatment, thus demonstrating that endocrine therapy resistance might occur^59–61^. Two additional patients with HER2-negative primary tumors had acquired *ERBB2* amplification in ctDNA at baseline sampling before combined treatment, thus suggesting clonal evolution toward a more aggressive subtype^62^. This finding may indicate that patients could benefit from anti-HER2 therapy. Another patient with triple-negative BC had *EGFR* amplification in ctDNA at baseline, and the copy number was highest when the disease progressed. According to ctDNA detection, patients with *EGFR* amplification may be considered for targeted therapy with lapatinib and may benefit from this approach^63^. Overall, these findings suggest that ctDNA detection might provide effective information for treatment guidance^64^.

Serial monitoring could be performed in only 8 patients. Dynamic changes in mutations and copy number variants in ctDNA can provide useful data in terms of response to treatment, particularly the association between *PIK3CA/TP53* mutation and tumor burden. This finding supports the results of previous studies^42,63^. Some variants not originally detected appeared during treatment, thus reflecting treatment resistance and/or clonal evolution^65^. A patient was identified to bear *PDGFRA* and *KIT* amplification when the disease progressed. Alterations in these genes are involved in *PDGF* signaling pathway activation, which is associated with tumor angiogenesis^66–68^. A previous study has shown that PDGFR expression may reflect *VEGF* signaling pathway resistance, and consequently that inhibition of *VEGFR-2* plus *PDGFR* induces tumor vessel regression^69,70^. In our study, no genetic variations were detected in 4 patients from baseline to progression until the last follow-up. A possible explanation is that alterations were not present within the detectable variants in our panel. Hence, we believe that whole-exome analysis of plasma ctDNA should be performed to explore heterogeneity and evolutionary cloning in BC to clarify the mechanism of drug resistance.

## Conclusions

In conclusion, this prospective study shows that apatinib combined with oral vinorelbine may enable good disease control in patients with HER2-negative mBC who previously received treatments that failed. Patients in better condition and those with delayed administration achieved longer PFS. The main AEs were similar to those of apatinib or vinorelbine alone, and were limited through use of appropriate care. Full oral administration was more acceptable, particularly in patients who had received intravenous therapy for a long time. To our knowledge, this is the first study demonstrating a potential clinical role of liquid biopsy applied to antiangiogenic combination therapy. The results of ctDNA target sequencing indicated that the mutation burden and VAF of ctDNA may be informative in antiangiogenic-based therapies. Patients with gene variants or higher VAF displayed poorer outcomes. A dynamic change in ctDNA might thus mirror gene clonal shifts, disease progression or resistance. However, the results of our ctDNA research remain to be further explored, owing to the small sample size in this study. Additional prospective studies with large randomized controlled trials may be needed to assess the long-term clinical benefits. Our results also implied that the resistance mechanism of angiogenic therapy, particularly in combination therapy, may be highly complicated. Further validation of our findings in a larger number of patients, with adequate control arms and comprehensive analysis of multiomics data, is needed.
