# Supplementary material for: Phase II study of apatinib in combination with oral vinorelbine in heavily pretreated HER2-negative metastatic breast cancer and clinical implications of monitoring ctDNA
Source: Cancer Biol Med. 2021 Aug 15;18(3):875–87. doi: 10.20892/j.issn.2095-3941.2020.0418 (PMC8330536; doi:10.20892/j.issn.2095-3941.2020.0418)
Supplement: Supplementary file 1 [file cbm-18-875-s001.pdf]

Supplementary materials

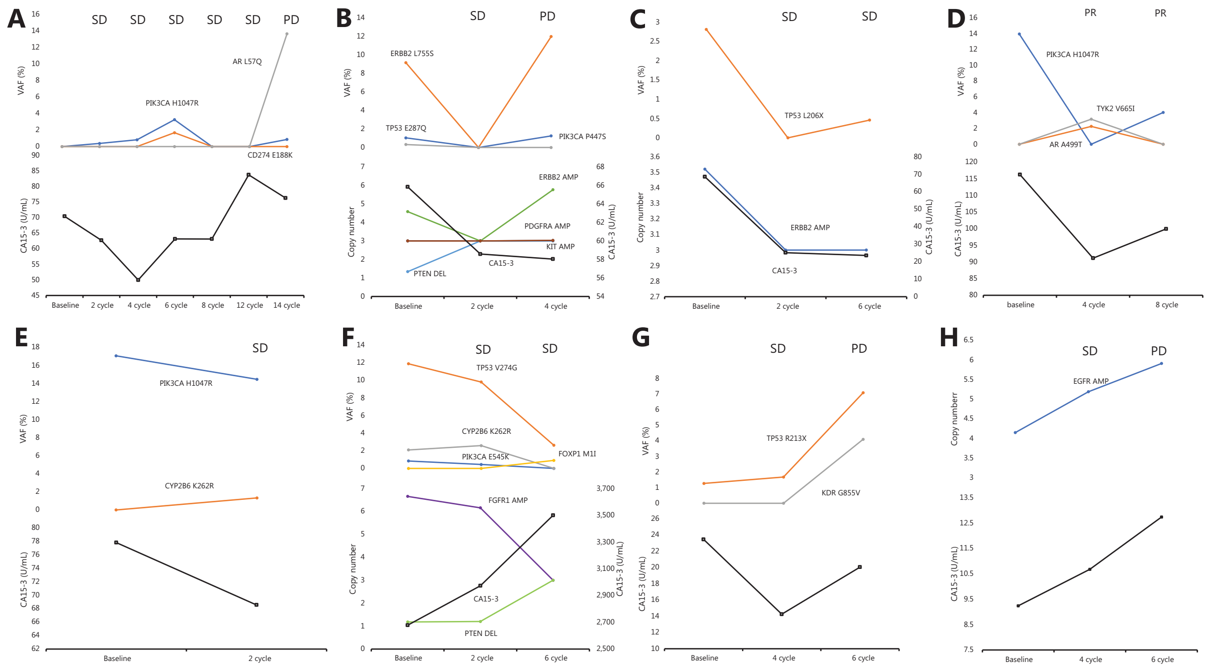

Figure S1 Serial monitoring of alterations in ctDNA from 8 patients with MBC. Graphs show VAF (%), copy number, and CA 15-3 (U/mL) dynamic alterations in patients A–H. (A) Patient 1 (HR positive, HER2 negative) was diagnosed at age 61 in 1996 and developed metastases in 2014. She received 2 lines of endocrine therapy (exemestane, toremifene) and then paclitaxel combined with carboplatin as chemotherapy, but treatment failed, and the disease worsened. She began to receive apatinib plus oral vinorelbine in August 2016 and had blood samples collected at baseline and during treatment. No variant was identified at baseline in the ctDNA. A low-frequency *PIK3CA* mutation was detected after 2 cycles of treatment after the response (SD), which then further increased and was accompanied by a subclonal mutation in CD274 after 6 cycles of treatment (SD). Mutations were lost after 8 cycles of treatment, when the disease was still clinically stable (SD). However, when disease progressed with the onset of brain metastasis after 14 cycles of treatment (PD), mutations in AR and subclonal mutations in PIK3CA were detected in ctDNA, thus mirroring the disease progression. There was no close correlation among CA15-3 concentration, mutation, and disease progression. (B) Patient 2 (HR positive, HER2 negative) was diagnosed with breast cancer in 2013 at age 36 and developed metastases in 2015. She received docetaxel and capecitabine followed by letrozole until visceral metastasis progressed. She began to receive apatinib plus oral vinorelbine in August 2016 and had blood samples collected at baseline and during treatment. Baseline ctDNA detection showed *ERBB2* amplification, *PTEN* deletion, and mutations in *TP53*, *PIK3CA*, and *ERBB2*. *ERBB2*, *TP53*, *PIK3CA*, and *PTEN* disappeared after a positive response after 2 cycles of treatment, whereas *ERBB2* and *TP53* variants were still detectable after 4 cycles of treatment after disease progression. *KIT* and *PDGFRA* amplifications were detected after disease progression, thus suggesting resistant disease. However, the CA15-3 concentration did not indicate a response to treatment. (C) Patient 3 (HR positive, HER2 negative) was diagnosed at age 59 in 2008 and developed metastases in 2014. She received 2 lines of endocrine therapy (exemestane plus everolimus, toremifene), and the disease worsened. She was switched to apatinib plus oral vinorelbine in November 2016 and had blood samples collected at baseline and during treatment. Amplified *ERBB2* and mutations in *TP53* were identified in ctDNA at baseline. She positively responded after 2 cycles of treatment (SD), and the variants disappeared. The *TP53* mutation was again detected at a low frequency after 6 cycles of treatment, when the disease was still stable (SD). However, the disease progressed after 10 cycles of treatment (PD), and no samples could be collected during this period. Although gene alterations were not predictive of disease progression, a similar trend of association of *TP53* mutation with tumor burden and CA15-3 concentration was observed. (D) Patient 4 (HR positive, HER2 negative) was diagnosed at age 65 in 2008 and developed metastases in 2014. She received docetaxel and capecitabine, followed by letrozole until visceral metastasis progressed. She was switched to apatinib plus oral vinorelbine and had blood samples collected at baseline in April 2017. Baseline ctDNA detection showed a mutation in *PIK3CA* (VAF 13.98%), which was lost after 4 cycles of treatment, concomitantly with a positive response to treatment, while mutations in *TYK2* and subclonal mutation in *AR* were first identified. *PIK3CA* mutation was again detected in ctDNA after 8 cycles of treatment, but to a lesser extent (i.e., VAF 4.03%). Mutations in *AR* and *TYK2* were lost when the disease maintained PR. The dynamic change in *PIK3CA* mutation suggested a clonal response. The change in

CA15-3 concentration paralleled the *PIK3CA* mutation. (E) Patient 5 (HR positive, HER2 negative) was diagnosed at age 61 in 2012 and developed metastases in 2016. She received chemotherapy followed by endocrine therapy (aromatase inhibitors), and the disease worsened. She received apatinib plus oral vinorelbine in September 2017 and had blood samples collected at baseline. Baseline ctDNA detection showed a mutation in *PIK3CA* (VAF 17.06%), whose frequency decreased (VAF 14.46%) after 2 cycles of treatment, after a positive response to treatment, and was accompanied by a mutation in *CYP2B6*, suggesting a clonal switch. The CA15-3 concentration decreased slightly after a positive disease response. (F) Patient 6 (HR positive, HER2 negative) was diagnosed at age 57 in 2015 and developed metastases in 2017. She received endocrine therapy (exemestane) and chemotherapy (paclitaxel, carboplatin, and gemcitabine), but the disease progressed. She was switched to apatinib plus oral vinorelbine in October 2017, and blood samples were collected at baseline. Baseline ctDNA analysis showed *FGFR1* amplification, *PTEN* deletion, and mutations in *TP53*, *PIK3CA*, and *CYP2B6*. After 2 cycles and 6 cycles of treatment associated with a positive disease response (SD), the frequency of *TP53*, *PIK3CA* and *CYP2B6* mutations decreased, the copy number of *FGFR1* also decreased, and the copy number of *PTEN* increased. *PIK3CA* mutation, *CYP2B6* mutation, *FGFR1* amplification and *PTEN* deletion disappeared after 6 cycles of treatment, whereas the *TP53* mutation frequency remained stable, thus suggesting a clonal response. The CA15-3 concentration increased and remained elevated. (G) Patient 7 (HR negative, HER2 negative) was diagnosed at age 57 in 2016 and developed metastases in 2017. She received 2 lines of chemotherapy, and the disease worsened. She was switched to apatinib plus oral vinorelbine in November 2017, and blood samples were collected at baseline. A mutation in *TP53* (VAF 1.27%) was detected at baseline in ctDNA, whose frequency slightly increased (VAF 1.68%) after 4 cycles of treatment after a positive disease response. However, the frequency increased more significantly (VAF 7.09%) when the disease progressed (PD) after 6 cycles of treatment, and was accompanied by a mutation in *KDR*, thus suggesting the development of resistant disease. The CA15-3 concentration was normal at baseline and during treatment, without significant changes. (H) Patient 8 (HR negative, HER2 negative) was diagnosed at age 62 in 2013 and developed metastases in 2015. She received docetaxel combined with capecitabine and was then recruited for a clinical trial of olaparib until disease progressed. She was switched to apatinib plus oral vinorelbine and had blood samples drawn at baseline in September 2016. Baseline ctDNA detection showed amplified *EGFR*. The disease was stable after 4 cycles of treatment (SD) but progressed after 6 cycles (PD). The copy number of *EGFR* amplification increased in parallel with disease progression. The CA15-3 concentration was initially normal but showed an increasing trend accompanied by *EGFR* amplification. Enhanced copy number of *EGFR* amplification and CA15-3 concentration suggested the development of resistant disease.

Table S1 Capture probes for 230 genes

ABCB1	*CREBBP*	*FGFR1*	*NF1*	*STAT5B*	*SEC31A*	*CXCR4*	*IFNL4*	*PPARD*	*XRCC1*
ATM	*CYP2E1*	*GOPC*	*PIK3CA*	*TYK2*	*APC*	*DNMT3A*	*LRIG3*	*RNF43*	*PPFIBP1*
CCDC6	*EPHA2*	*JAK3*	*RAC1*	*CDK5RAP2*	*BTK*	*ESR1*	*MTHFR*	*SLCO1B1*	*DCTN1*
CHST3	*FES*	*MET*	*SDC4*	*PCM1*	*CDK4*	*FLT3*	*NQO1*	*TP53*	*C2orf44*
CYP2D6	*GNAS*	*NCOA4*	*STAT3*	*VCL*	*CTNNB1*	*IFNL3*	*PMS2*	*XPC*	*ATIC*
EML4	*JAK2*	*PDGFRB*	*TUBB1*	*ALK*	*DDR2*	*SH2B3*	*RIT1*	*MYO5A*	*CBL*
FDPS	*MAP3K5*	*PTPN11*	*ETV6*	*BRCA2*	*ERCC2*	*MSH6*	*SLC34A2*	*CLTC*	*CDKN2B*
GNAQ	*NAT2*	*RRM2B*	*KTN1*	*CDH1*	*FIP1L1*	*NPM1*	*TET2*	*CARS*	*CYP2C8*
JAK1	*PDGFRA*	*SPG7*	*TPM4*	*CTLA4*	*IDH2*	*PML*	*UMPS*	*ARID2*	*EGFR*
MAP2K2	*PTEN*	*TSC2*	*AKT3*	*DCK*	*KRAS*	*RHOA*	*KIAA1598*	*CASP7*	*FBXW7*
MYD88	*RRM2*	*RPL13*	*BRCA1*	*ERCC1*	*MSH2*	*SLC22A12*	*FGFR1OP*	*CDKN2A*	*GNA11*
PAK5	*SOD2*	*HOOK3*	*CDA*	*FGFR4*	*NOTCH2*	*TEKT4*	*FN1*	*CYP1B1*	*IMPDH2*
PTCH1	*TSC1*	*TFG*	*CSF3R*	*IDH1*	*PLCG2*	*UGT1A8*	*ARID1A*	*EGF*	*MAP2K1*
RRM1	*GSTA1*	*AKT2*	*CYP4B1*	*KIT*	*RET*	*ERC1*	*CARD11*	*F3*	*MTRR*
SMO	*GOLGA5*	*BRAF*	*ERBB4*	*MPL*	*SLC19A1*	*TRIM33*	*CDKN1B*	*GATA3*	*NUDT15*
TRRAP	*SQSTM1*	*CD79B*	*FGFR3*	*NOTCH1*	*SULT2B1*	*MSN*	*CYP1A1*	*IL7R*	*PRKAR1A*
TYMS	*XRCC5*	*CSF1R*	*HRAS*	*PIK3R2*	*UGT1A1*	*ARAF*	*DYNC2H1*	*LTK*	*ROS1*
ZCCHC8	*AKT1*	*CYP3A5*	*KIF5B*	*RAF1*	*CLIP1*	*CALR*	*EZR*	*MTR*	*SMAD4*
KLC1	*BCR*	*ERBB3*	*MLL3*	*SF3B1*	*TRIM27*	*CDKN1A*	*GALNT14*	*NTRK1*	*TPMT*
RARA	*CD74*	*FGFR2*	*NOS3*	*STK11*	*RANBP2*	*CYP19A1*	*IL10*	*PRKACA*	*ZRSR2*
ABL1	*CRLF2*	*GSTP1*	*PIK3R1*	*U2AF1*	*AR*	*DPYD*	*LRRK2*	*ROCK1*	*PWWP2A*
AXIN1	*CYP3A4*	*KDR*	*RAD50*	*STRN*	*C8orf34*	*EZH2*	*MTOR*	*SLCO1B3*	*HIP1*
CCND3	*ERBB2*	*MLH1*	*SEMA3C*	*TRIM24*	*CDK6*	*FRK*	*NRAS*	*TPM3*	*HLA-A*

Table S2 Treatments with different initial apatinib doses

	500 mg/day (*n* = 17) *n* (%)		425 mg/day (*n* = 23) *n* (%)	Total (*n* = 40) *n* (%)
Delayed administration	12 (70.6)		15 (65.2)	27 (67.5)
Apatinib dose modification	8 (47.1)		9 (39.1)	17 (42.5)
Apatinib settled dose	425 mg (5 cases)	250 mg (3 cases)	250 mg (9 cases)	
Vinorelbine dose modification	8 (47.1)		8 (34.8)	16 (40.0)
Discharged	3 (17.6)		2 (8.7)	5 (12.5)

Table S3 Adverse events by apatinib initial dose

Adverse events	500 mg/day (n = 17)			425 mg/day (n = 23)		
	All grades	Grade 1/2	Grade 3/4	All grades	Grade 1/2	Grade 3/4
	(%)	(%)	(%)	(%)	(%)	(%)
Myelosuppression (hematology)	12 (70.6)	9 (52.9)	3 (17.6)	15 (65.2)	12 (52.2)	3 (13.0)
Leukopenia	10 (58.8)	10 (58.8)	0 (0)	12 (52.2)	10 (43.5)	2 (8.7)
Neutropenia	11 (64.7)	8 (47.1)	3 (17.6)	11 (47.8)	9 (39.1)	2 (8.7)
Thrombocytopenia	6 (35.3)	5 (29.4)	1 (5.9)	4 (17.4)	4 (17.4)	0 (0)
Decreased hemoglobin	5 (29.4)	5 (29.4)	0 (0)	4 (17.4)	4 (17.4)	0 (0)
Gastrointestinal reaction	11 (64.7)	5 (29.4)	6 (35.3)	17 (73.9)	14 (60.9)	3 (13.0)
Nausea	8 (47.1)	7 (41.2)	1 (5.9)	15 (65.2)	15 (65.2)	0 (0)
Diarrhea	9 (52.9)	6 (35.3)	3 (17.6)	10 (43.5)	7 (30.4)	3 (13.0)
Vomiting	5 (29.4)	3 (17.6)	2 (11.8)	7 (30.4)	7 (30.4)	0 (0)
Hypertension	11 (64.7)	5 (29.4)	6 (35.3)	14 (60.9)	10 (43.5)	4 (17.4)
Pain	10 (58.8)	8 (47.1)	2 (11.8)	14 (60.9)	11 (47.8)	3 (13.0)
Malaise	8 (47.1)	7 (41.2)	1 (5.9)	13 (56.5)	12 (52.2)	1 (4.3)
Anorexia	9 (52.9)	8 (47.1)	1 (5.9)	11 (47.8)	11 (47.8)	0 (0)
Elevated transaminase	9 (52.9)	9 (52.9)	0 (0)	10 (43.5)	10 (43.5)	0 (0)
Hand-foot skin reaction	10 (58.8)	8 (47.1)	2 (11.8)	9 (39.1)	8 (34.8)	1 (4.3)
Proteinuria	9 (52.9)	8 (47.1)	1 (5.9)	6 (26.1)	6 (26.1)	0 (0)
Elevated bilirubin	7 (41.2)	7 (41.2)	0 (0)	6 (26.1)	5 (21.7)	1 (4.3)
Mucositis	5 (29.4)	4 (23.5)	1 (5.9)	6 (26.1)	4 (17.4)	2 (8.7)
Hemorrhage	5 (29.4)	4 (23.5)	1 (5.9)	3 (13.0)	3 (13.0)	0 (0)
Sinus tachycardia	3 (17.6)	3 (17.6)	0 (0)	3 (13.0)	3 (13.0)	0 (0)
Elevated creatinine	2 (11.8)	2 (11.8)	0 (0)	1 (4.3)	1 (4.3)	0 (0)

Table S4 Major adverse events and delayed administration timeline

Onset of adverse events	Median time (day)					
	Apatinib initial dose 500 mg/day		Apatinib initial dose 425 mg/day		Total	
	All grades	Grade 3/4	All grades	Grade 3/4	All grades	Grade 3/4
Hypertension	2.0 (0–20)	1.0 (0–2)	3.5 (1–49)	2.5 (1–4)	3.0 (0–49)	1.5 (0–4)
Proteinuria	15.0 (7–53)	15.0 (15–15)	29.5 (5–52)	–	19.0 (5–53)	15.0 (15–15)
Hand-foot skin reaction	20.5 (5–90)	22.0 (14–30)	34.5 (21–80)	28.0 (28–28)	28.0 (5–90)	28.0 (14–30)
Mucositis	29.0 (7–38)	28.0 (28–28)	25.0 (20–60)	24.0 (20–28)	28.0 (7–60)	28.0 (20–28)
Myelosuppression	11.5 (3–60)	7.0 (3–15)	15.0 (3–28)	17.5 (11–24)	14.5 (3–60)	11.0 (3–24)
Gastrointestinal reaction	7.0 (1–65)	8.5 (3–65)	10.0 (1–33)	14.0 (13–33)	8.5 (1–65)	13.0 (3–65)
All	2.5 (0–29)	2.0 (0–65)	3.5 (1–23)	4.0 (1–28)	3.5 (0–29)	2.5 (0–65)
First time of delayed administration	2.5 (0–65)		8.0 (1–80)		5.0 (0–80)	
First time of dose modification	13.0 (3–72)		14.0 (4–83)		14.0 (3–83)	

Table S5 Efficacy by number of treatment lines and hormone receptor status

Efficacy characteristics (*n*, %)	2nd line (*n* = 20)	3rd line/above (*n* = 15)	HR positive (*n* = 17)	HR negative (*n* = 18)
PR	4 (20.0)	2 (13.3)	2 (11.8)	4 (22.2)
SD	12 (60.0)	11 (73.3)	12 (70.6)	11 (61.1)
PD	4 (20.0)	2 (13.3)	3 (17.6)	3 (16.7)
ORR	4 (20.0)	2 (13.3)	2 (11.8)	4 (22.2)
CBR	10 (50.0)	6 (40.0)	7 (41.2)	9 (50.0)
mPFS (m, range)	4.8 (1.1–8.6)	5.2 (4.0–6.4)	4.4 (2.0–6.8)	5.2 (3.3–7.1)
mOS (m, range)	14.7 (8.9–20.5)	27.0 (10.5–43.6)	22.1 (7.7–36.6)	15.8 (10.1–21.5)

PR, partial remission; SD, stable disease; PD, progressive disease; ORR, objective response rate; CBR, clinical benefit rate; mPFS, median progression free survival; mOS, median overall survival; HR, hormone receptor.

Table S6 Multivariate Cox proportional hazard models predicting OS for patients receiving combined therapy

Variables	HR (95% CI)	*P*
Age (< 55/≥ 55)	0.574 (0.217–1.520)	0.264
Hormone receptor (neg/pos)	0.887 (0.412–1.908)	0.758
ECOG PS (0/1)	0.484 (0.147–1.595)	0.233
Apatinib initial dose (425 mg/500 mg)	0.970 (0.300–3.142)	0.960
Proteinuria (no/yes)	1.504 (0.614–3.682)	0.372
Delayed administration (no/yes)	1.600 (0.594–4.312)	0.353
Chest wall recurrence (no/yes)	0.377 (0.160–0.886)	0.025

OS, overall survival; ECOG PS, Eastern Cooperative Oncology Group Performance Status.

Table S7 Patient characteristics for ctDNA detection at baseline

Characteristics	*n* (%)
Age, years	
< 55	10 (50.0)
≥ 55	10 (50.0)
Hormone receptor	
Negative	8 (40.0)
Positive	12 (60.0)
Histopathologic grade	
I–II	9 (45.0)
III	8 (40.0)
Unknown	3 (15.0)
Tumor size (cm)	
≤ 2.0	5 (25.0)
> 2.0	12 (60.0)
Unknown	3 (15.0)
Axillary lymph node metastasis	
Positive	16 (80.0)
Negative	3 (15.0)
Unknown	1 (5.0)
TNM stage	
I–II	9 (45.0)
III	8 (40.0)
Unknown	3 (15.0)
Visceral metastasis	
Yes	11 (55.0)
No	9 (45.0)
Metastasis sites	
≥ 3	11 (55.0)
< 3	9 (45.0)
Lines of treatment	
< 3 line	11 (55.0)
≥ 3 line	9 (45.0)

Table S8 Variants in ctDNA tracking in 20 patients with metastatic breast cancer, detected by NGS

Patient ID	Study sample, cycles	SNV, VAF			Gene amp*	Gene del*
		Gene	Mutation	VAF, %		
1	Baseline					
	2					
	4					
	6					
	8					
2	Baseline					
	2	*PIK3CA*	*H1047R*	0.38		
	4	*PIK3CA*	*H1047R*	0.82		
	6	*PIK3CA*	*H1047R*	3.25		
		CD274	*E188K*	1.68		
	8					
	12					
	14	*PIK3CA*	*H1047R*	0.86		
		AR	*L57Q*	13.63		
3	Baseline					
	4					
	6					
	8					
	10					
4	Baseline					
	2					
5	Baseline					
	2					
	4					
	6					
	8					
6	Baseline	*TP53*	*E287Q*	1.04	*ERBB2*	*PTEN*
		ERBB2	*L755S*	9.15		
		PIK3CA	*P447S*	0.32		
	2					
	4	*TP53*	*E287Q*	1.25	*ERBB2*	
		ERBB2	*L755S*	11.97	*PDGFRA*	
					KIT	
7	Baseline	*TP53*	*L206X*	2.81	*ERBB2*	
	2					
	6	*TP53*	*L206X*	0.46		
8	Baseline	*PIK3CA*	*H1047R*	13.98		
	4	*AR*	*A499T*	2.27		
		TYK2	*V665I*	3.18		
	8	*PIK3CA*	*H1047R*	4.03		
9	Baseline	*TP53*	*R248Q*	3.44		
		CDKN2A	*A127T*	3.25		
10	Baseline	*PIK3CA*	*H1047R*	17.06		
	2	*PIK3CA*	*H1047R*	14.46		
		CYP2B6	*K262R*	1.33		
11	Baseline	*PIK3CA*	*E545K*	0.85	*FGFR1*	*PTEN*
		TP53	*V274G*	11.88		
		CYP2B6	*K262R*	2.1		
	2	*PIK3CA*	*E545K*	0.45	*FGFR1*	*PTEN*
		TP53	*V274G*	9.81		
		CYP2B6	*K262R*	2.59		
	6	*TP53*	*V274G*	2.63		
		FOXP1	*M1I*	0.91		
12	Baseline	*TP53*	*R213X*	1.27		
	4	*TP53*	*R213X*	1.68		
	6	*TP53*	*R213X*	7.09		
		KDR	*G855V*	4.1		
13	Baseline	*PIK3CA*	*H1047R*	0.7		
14	Baseline					
15	Baseline	*TP53*	*S215I*	9.28		
		ERCC1	*D129N*	8.44		
		AXIN1	*A185T*	2.13		
16	Baseline					
17	Baseline					*PTEN*
18	Baseline	*ESR1*	*Y537C*	1.4		
		BRCA1	*S643C*	1.4		
19	Baseline				*EGFR*	
	4				*EGFR*	
	6				*EGFR*	
20	Baseline	*ALK*	*F921L*	3.81	*FGFR1*	
		AKT1	*E17K*	34.22		
		TP53	*R175H*	31.83		

Copy number > 3 and < 1.5 were determined as amplification (amp*) and deletion (del*), respectively. SNV, single nucleotide variants; VAF, variant allele frequency.

Supplementary material 1

Inclusion criteria

1. Women 18–75 years old and HER2 negative (immunohistochemistry or fluorescence *in situ* hybridization);
2. ECOG PS score: 0–1, expected survival time ≥ 3 months;
3. Pathologically or cytologically confirmed breast cancer;
4. Anthracycline-/taxane- pretreated (adjuvant, neoadjuvant) breast cancer and failure of 1–2 standard chemotherapies after recurrence and metastasis;
5. At least ≥ 1 measurable lesion according to RECIST 1.1;
6. Sufficient organ function; laboratory test indexes complying with the following requirements:

   Blood: neutrophils ≥ 1.5 G/L, platelet count ≥ 80 G/L, hemoglobin ≥ 90 g/L.

   Liver function: serum bilirubin ≤ 1.5 times the upper limit of normal; ALT and AST ≤ 2.5 times the upper limit of normal; ALT and AST ≤ 5 times the upper limit of normal in the presence of liver metastasis.

   Renal function: serum creatinine ≤ 1.0 times the upper limit of normal, creatinine clearance > 50 mL/min (Cockcroft-Gault formula).
7. For women of child-bearing age, negative test for pregnancy (serum or urine) within 7 days before recruitment and willingness to use the appropriate methods of contraception during the trial and 8 weeks after the last administration;
8. Ability to swallow oral drugs;
9. Good compliance with the therapy and follow-up to be scheduled, and ability to understand the study protocol and sign the informed consent form.

Exclusion criteria

1. Pregnancy or lactation growth period; failure to take effective contraception;
2. Administration of ≥ 3 chemotherapies (not including endocrine therapy) after recurrence and metastasis; involvement in other clinical trials 4 weeks before the start of the study;
3. A variety of factors affecting the oral administration and absorption of drugs;
4. Previous administration of anti-VEGF of anti-VEGFR therapies;
5. Rapid progression of viscera invasion (liver lesion > 1/2 viscera area or liver dysfunction);
6. Uncontrollable mental illness;
7. Serious adverse events to oral vinorelbine or allergic reaction to vinorelbine;
8. Only bone metastasis without other measurable lesions;
9. Severe cardiovascular diseases;
10. Severe upper gastrointestinal ulcer or malabsorption syndrome;
11. Abnormal bone marrow function (neutrophils < 1.5 G/L, platelet count < 75 G/L, hemoglobin < 90 g/L);
12. Abnormal renal function (serum creatinine > 1.5 times the upper limit of normal);
13. Abnormal liver function (serum bilirubin ≥ 1.5 times the upper limit of normal);
14. Uncontrollable brain metastasis;
15. Poor compliance with the therapy.

Supplementary material 2

Library preparation and NGS

Peripheral blood samples of patients were collected (10 mL) in STRECK vacutainer tubes, and plasma was separated by centrifugation. A circulating nucleic acid kit (Qiagen, Hilden, Germany) was used according to the manufacturer's protocols. DNA was quantified with a Nanodrop 2000 instrument (Thermo Fisher) and the Qubit dsDNA high sensitivity assay (Thermo Fisher). Sequencing libraries were constructed according to the Illumina standard library construction instructions (Illumina, San Diego, CA, USA). The various libraries were then hybridized with 230 gene (**Supplementary Table S1**) capture probes, which enriched for the coding regions and selected introns of genes with known relevance to BC, including common hallmarks of cancer, particularly the *VEGF* signaling pathway. The target-enriched libraries were then pooled and sequenced on the Illumina HiSeq X Ten NGS platform (Illumina).

Bioinformatics analysis

Pre-processing of raw sequence data and quality control statistics were performed by using an in-house QC tool. Reads were aligned to the GRCh37 version of the human genome with Burrows-Wheeler Aligner software (BWA, version 0.5.9). PCR duplicates were marked with the MarkDuplicates tool

in Picard. Indel Realigner and Base Recalibrator in Genome Analysis Toolkit (GATK; version 2.7) were used for realignment and recalibration of the BWA alignment results, respectively. The mutect2 algorithm was used for identifying the paired-sample variant calling of single nucleotide variants and insertions/deletions on tumor and matched normal samples. All variants were annotated with Annovar. Copy number variants and fusion calling were performed with the corresponding in-house algorithms. Captured DNA fragments were sequenced on an Illumina HiSeq X Ten as paired-end 150-bp reads. To ensure the quality of data, the following criteria were applied to filter raw variant results: all reads were filtered by high mapping quality (≥ 30) and base quality (≥ 30); the mutant reads were required to be supported by positive and negative strands; the average effective sequencing depth on target per sample was required to be $\geq 800\times$ (cfDNA).
